# Supplementary material for: Secretory quality control constrains functional selection-associated protein structure innovation
Source: Commun Biol. 2022 Mar 25;5:268. doi: 10.1038/s42003-022-03220-3 (PMC8956723; doi:10.1038/s42003-022-03220-3)
Supplement: Supplementary file 2 — Supplementary Information [file 42003_2022_3220_MOESM2_ESM.pdf]

Supplementary Table 1. Mutants examined for their effects on cellular folding of human CRP

Step 1 mutants *	Step 2 mutants #
C36S/H38C/H95C/C97S, strands C-H ξ	N61A, calcium binding site mutant ξ
V35C/C36S/C97S/T98C, strands C-H	N61Q, calcium binding site mutant ξ
C36H/H38C/H95C/C97H, strands C-H	N61S, calcium binding site mutant ξ
V35C/C36H/C97H/T98C, strands C-H	Q139A, calcium binding site mutant ξ
C36S/H38C/H95C/C97S/Y175R, strand C-H	E138A, calcium binding site mutant ξ
C36S/W110C, strands H-I ξ	E138Q, calcium binding site mutant ξ
C36S/C97S/S99C/E108C, strands H-I ξ	D140A, calcium binding site mutant ξ
C36S/I63C/V77C/C97S, strands E-F ξ	C36S/C97S, disulfide bond mutant ξ
C36S/S53C/L64C/C97S, strands D-E ξ	
C36S/T76C/E81C/C97S, strands F-G ξ	

* In all mutants, a disulfide bond was introduced between two adjacent strands in the spontaneously formed core of strands C to I guided by the crystal structure of human CRP. The wildtype intra-subunit disulfide bond (Cys36-Cys97, strands C-H) was also mutated. With such design, each mutant harbors 3 to 4 mutations within the core, which may further corroborate the robustness of the step 1 folding. The correct folding of the core was denoted by the efficient formation of the introduced disulfide bonds [1].

# The correct folding of the native structures was determined by conformation-specific ELISA of the cultured media [1].

ξ Mutants have been prepared and examined in our previous work [1].

Supplementary Table 2. Mutants examined for their effects on secretion of human CRP

E. coli mutants	COS-7 mutants
N61A, calcium binding site mutant ξ	N61A, calcium binding site mutant ξ
Q150A, calcium binding site mutant ξ	Q150A, calcium binding site mutant ξ
E138A, calcium binding site mutant ξ	E138A, calcium binding site mutant ξ
D140A, calcium binding site mutant ξ	D140A, calcium binding site mutant ξ
D140N, calcium binding site mutant ξ	Q139A, calcium binding site mutant ξ
C36S/C97S, disulfide bond mutant	C36A/C97A, disulfide bond mutant ξ
D155A, pentamer assembly mutant	D155A, pentamer assembly mutant
R118A, pentamer assembly mutant	R118A, pentamer assembly mutant
P12C/K119C, pentamer assembly mutant	P12C/K119C, pentamer assembly mutant
R118C/D155C, pentamer assembly mutant	R118C/D155C, pentamer assembly mutant
E101A/D155A/E197A, pentamer assembly mutant	E101Q/D155N/E197Q, pentamer assembly mutant
Δ168-176, truncation mutant ξ	Δ168-176, truncation mutant
C36S/H38C/H95C/C97S, disulfide bond mutant ξ	Δ1-31, truncation mutant
V35C/C36S/C97S/T98C, disulfide bond mutant	Δ1-54, truncation mutant
C36S/S53C/L64C/C97S, disulfide bond mutant ξ	Δ180-206, truncation mutant
C36S/F66C/S74C/C97S, disulfide bond mutant	Δ199-206, truncation mutant
C36S/I63C/V77C/C97S, disulfide bond mutant ξ	
C36S/T76C/E81C/C97S, disulfide bond mutant ξ	
C36S/G72C/E85C/C97S, disulfide bond mutant	
C36H/H38C/H95C/C97H, disulfide bond mutant	
V35C/C36H/C97H/T98C, disulfide bond mutant	
C36S/H38C/H95C/C97S/Y175R, disulfide bond mutant	
V35C/C36S/C97S/T98C/I174R, disulfide bond mutant	
C36S/W110C, disulfide bond mutant ξ	
C36S/C97S/S99C/E108C, disulfide bond mutant ξ	

C36S/T56C/C97S/A131C, disulfide bond mutant ξ

C36S/Y49C/C97S/Q150C, disulfide bond mutant ξ

C36S/T41C/C97S/G154C, disulfide bond mutant ξ

C36S/L37C/C97S/V159C, disulfide bond mutant ξ

Y49C/Q150C, disulfide bond mutant

T41C/G154C, disulfide bond mutant

K7C/C36S/C97S/P202C, disulfide bond mutant

F9C/C36S/C97S/T200C, disulfide bond mutant ξ

S18C/C36S/97S/G196C, disulfide bond mutant ξ

S21C/C36S/97S/E193C, disulfide bond mutant ξ

C36S/C97S/E108C/M161C, disulfide bond mutant ξ

S21C/C36S/S53C/C97S, disulfide bond mutant ξ

A8C/C36S/H38C/C97S, disulfide bond mutant ξ

C36S/V86C/C97S/S149C, disulfide bond mutant ξ

A8C/C36S/C97S/G157C, disulfide bond mutant ξ

V10C/C36S/C97S/D155C, disulfide bond mutant ξ

S21C/C36S/C97S/I134C, disulfide bond mutant ξ

S15C/C36S/C97S/G148C, disulfide bond mutant ξ

K23C/C36S/C97S/S132C, disulfide bond mutant ξ

ξ Mutants have been prepared and examined in our previous work [1].

Supplementary Table 3. Intrinsically disordered proteins with disordered content > 50 %

Protein *	Organism	Disordered content	Signal peptide
Transcriptional and immune response regulator	Human	100.00 %	No
Cytochrome c oxidase copper chaperone	Human	100.00 %	No
Isoform Tau-F of Microtubule-associated protein tau	Human	100.00 %	No
Stathmin	Human	100.00 %	No
Cyclin-dependent kinase inhibitor 1B	Human	100.00 %	No
Eukaryotic translation initiation factor 4E-binding protein 1	Human	100.00 %	No
Gamma-synuclein	Human	100.00 %	No
Isoform 5 of Myelin basic protein	Human	100.00 %	No
Non-histone chromosomal protein HMG-17	Human	100.00 %	No
Nuclear protein 2	Human	100.00 %	No
Cyclin-dependent kinase inhibitor 1	Human	100.00 %	No
Isoform tumor suppressor ARF of Tumor suppressor ARF	Human	100.00 %	No
Isoform Crk-I of Adapter molecule crk	Human	100.00 %	No
Alpha-synuclein	Human	100.00 %	No
C-C motif chemokine 28	Human	100.00 %	Yes
Calreticulin	Human	100.00 %	Yes
High mobility group protein HMG-I/HMG-Y	Human	100.00 %	No
Thymosin beta-4	Human	100.00 %	No
Bone sialoprotein 2	Human	100.00 %	Yes
cAMP-dependent protein kinase inhibitor alpha	Human	100.00 %	No
High mobility group protein HMGI-C	Human	100.00 %	No
Prothymosin alpha	Human	100.00 %	No
Osteopontin	Human	100.00 %	Yes

Huntingtin-interacting protein K	Human	100.00 %	No
Brain acid soluble protein 1	Human	100.00 %	No
Involucrin	Human	100.00 %	No
Calmodulin regulator protein PCP4	Human	100.00 %	No
Alpha-S1-casein	Human	100.00 %	Yes
Eukaryotic translation initiation factor 4E-binding protein 2	Human	100.00 %	No
Humanin	Human	100.00 %	No
Neuromodulin	Human	100.00 %	No
RING1 and YY1-binding protein	Human	100.00 %	No
Cyclin-dependent kinase inhibitor 1C	Human	100.00 %	No
Securin	Human	100.00 %	No
von Hippel-Lindau disease tumor suppressor	Human	100.00 %	No
Beta-synuclein	Human	100.00 %	No
PCNA-associated factor	Human	100.00 %	No
E3 ubiquitin-protein ligase PPP1R11	Human	100.00 %	No
Nuclear protein 1	Human	100.00 %	No
P antigen family member 4	Human	100.00 %	No
Nucleolar and coiled-body phosphoprotein 1	Human	100.00 %	No
Achaete-scute homolog 1	Human	100.00 %	No
RNA-binding protein FUS	Human	96.39 %	No
Basic salivary proline-rich protein 4	Human	94.84 %	Yes
Protein max	Human	93.13 %	No
Isoform A of Reticulon-4	Human	91.36 %	No
Reticulon-4	Human	88.00 %	No
Metastasis-suppressor KiSS-1	Human	86.23 %	Yes
Breast cancer type 1 susceptibility protein	Human	82.88 %	No
Polyglutamine-binding protein 1	Human	82.64 %	No

U1 small nuclear ribonucleoprotein 70 kDa	Human	81.01 %	No
Methyl-CpG-binding protein 2	Human	80.86 %	No
Selenocysteine insertion sequence-binding protein 2	Human	79.51 %	No
F-box only protein 5	Human	78.97 %	No
Homeobox protein Nkx-3.1	Human	78.63 %	No
Linker for activation of T-cells family member 1	Human	78.63 %	No
AF4/FMR2 family member 4	Human	77.39 %	No
Isoform 3 of REST corepressor 3	Human	75.95 %	No
Emerin	Human	73.62 %	No
REST corepressor 1	Human	72.58 %	No
Anaphase-promoting complex subunit CDC26	Human	70.59 %	No
Isoform 6 of LIM domain-binding protein 3	Human	70.32 %	No
Protein chibby homolog 1	Human	69.84 %	No
Abl interactor 2	Human	69.79 %	No
Statherin	Human	69.35 %	Yes
CSTB protein	Human	68.37 %	No
Nucleophosmin	Human	68.37 %	No
Peroxisomal biogenesis factor 19	Human	67.56 %	No
Transcription initiation factor TFIID subunit 6	Human	67.50 %	No
Protein Tob1	Human	66.67 %	No
Progesterone receptor	Human	63.34 %	No
Histatin-3	Human	62.75 %	Yes
Small proline-rich protein 2E	Human	62.50 %	No
Isoform B of Reticulon-4	Human	62.47 %	No
SOSS complex subunit C	Human	59.62 %	No
Negative elongation factor E	Human	59.21 %	No
Peroxisome proliferator-activated receptor	Human	59.21 %	No

gamma			
SOSS complex subunit B1	Human	58.29 %	No
Sclerostin	Human	58.22 %	Yes
CD166 antigen	Human	57.98 %	Yes
Histone H1.0	Human	57.73 %	No
Natriuretic peptides B	Human	57.46 %	Yes
Myelin basic protein	Human	56.25 %	No
ATM interactor	Human	56.14 %	No
Zinc finger protein 593	Human	55.22 %	No
Protein ELYS	Human	55.12 %	No
Anaphase-promoting complex subunit 15	Human	53.72 %	No
Ezrin	Human	52.90 %	No
Cyclin-dependent kinase 2-associated protein 1	Human	52.17 %	No
Apoptosis-stimulating of p53 protein 2	Human	52.13 %	No
Ataxin-3	Human	52.08 %	No
Signal recognition particle receptor subunit alpha	Human	51.88 %	No
Vesicle-associated membrane protein-associated protein B/C	Human	51.44 %	No
Parathyroid hormone-related protein	Human	51.41 %	Yes
Replication protein A 32 kDa subunit	Human	51.11 %	No
Peroxisomal targeting signal 1 receptor	Human	50.70 %	No
Surfeit locus protein 6	Human	50.42 %	No
Potassium voltage-gated channel subfamily E member 1	Human	50.39 %	No
Cell cycle regulator of non-homologous end joining	Mouse	100.00 %	No
RWD domain-containing protein 1	Mouse	100.00 %	No
Isoform 5 of Myelin basic protein	Mouse	100.00 %	No
Cellular retinoic acid-binding protein 1	Mouse	100.00 %	No

Bcl-2-modifying factor	Mouse	100.00 %	No
Myristoylated alanine-rich C-kinase substrate	Mouse	100.00 %	No
Protein phosphatase inhibitor 2	Mouse	100.00 %	No
Bcl2-associated agonist of cell death	Mouse	100.00 %	No
Protein LBH	Mouse	100.00 %	No
POU domain class 2-associating factor 1	Mouse	100.00 %	No
Protein B-Myc	Mouse	98.82 %	No
Osteopontin	Mouse	94.56 %	Yes
SPARC	Mouse	94.37 %	Yes
Protein BEX1	Mouse	89.06 %	No
Calsenilin	Mouse	75.00 %	No
Smoothelin-like protein 1	Mouse	74.29 %	No
Histone H1.0	Mouse	65.98 %	No
Bcl-2-like protein 11	Mouse	57.65 %	No
Sclerostin	Mouse	56.87 %	Yes
Protein ELYS	Mouse	54.66 %	No
Vesicle-associated membrane protein 4	Mouse	50.35 %	No
Ribonuclease H2 subunit B	Mouse	50.00 %	No
Synaptosomal-associated protein 25	Rat	100.00 %	No
Ermin	Rat	100.00 %	No
PRKC apoptosis WT1 regulator protein	Rat	100.00 %	No
Histone H1.4	Rat	99.09 %	No
Neuroendocrine protein 7B2	Rat	88.57 %	Yes
Vesicle-associated membrane protein 2	Rat	75.86 %	No
Epsin-1	Rat	75.13 %	No
Seminal vesicle secretory protein 4	Rat	62.50 %	Yes
Clathrin coat assembly protein AP180	Rat	62.19 %	No
Choline-phosphate cytidylyltransferase A	Rat	61.58 %	No
Caskin-1	Rat	57.90 %	No

Protein SIC1	Yeast	100.00 %	No
Nucleoporin NUP2	Yeast	100.00 %	No
Histone H2A.Z-specific chaperone CHZ1	Yeast	100.00 %	No
Protease A inhibitor 3	Yeast	100.00 %	No
Protein GIR2	Yeast	100.00 %	No
26S proteasome complex subunit SEM1	Yeast	100.00 %	No
Proteasome maturation factor UMP1	Yeast	100.00 %	No
Pre-mRNA-splicing factor NTR2	Yeast	100.00 %	No
Ribonucleotide reductase inhibitor protein SML1	Yeast	100.00 %	No
EKC/KEOPS complex subunit GON7	Yeast	100.00 %	No
60S acidic ribosomal protein P1-alpha	Yeast	100.00 %	No
12 kDa heat shock protein	Yeast	88.99 %	No
rRNA biogenesis protein RRP5	Yeast	81.43 %	No
Protein SAN1	Yeast	80.98 %	No
Synaptobrevin homolog 1	Yeast	79.49 %	No
Synaptobrevin homolog 2	Yeast	79.13 %	No
Transcription factor BYE1	Yeast	77.44 %	No
Nucleoporin NSP1	Yeast	73.75 %	No
DNA-directed RNA polymerase I subunit RPA49	Yeast	72.53 %	No
Nucleoporin NUP1	Yeast	72.21 %	No
Vacuolar protein-sorting-associated protein 36	Yeast	69.79 %	No
Nuclear mRNA export protein SAC3	Yeast	68.64 %	No
H/ACA ribonucleoprotein complex subunit NOP10	Yeast	67.24 %	No
Nucleoporin NUP100/NSP100	Yeast	66.74 %	No
Autophagy-related protein 13	Yeast	63.82 %	No
Ran-specific GTPase-activating protein 2	Yeast	62.08 %	No
DNA-directed RNA polymerase I subunit	Yeast	61.31 %	No

RPA14			
Transcription initiation factor IIE subunit alpha	Yeast	59.75 %	No
Pre-mRNA-splicing factor ISY1	Yeast	59.57 %	No
Suppressor protein STM1	Yeast	58.97 %	No
Dolichyl-diphosphooligosaccharide--protein glycosyltransferase subunit 3	Yeast	55.14 %	Yes
Regulator of Ty1 transposition protein 102	Yeast	54.78 %	No
Ubiquitin carboxyl-terminal hydrolase 10	Yeast	53.28 %	No
Centromere DNA-binding protein complex CBF3 subunit A	Yeast	52.20 %	No
KRR1 small subunit processome component homolog	Drosophila	100.00 %	No
Histone H4	Drosophila	78.64 %	No
Homeotic protein ultrabithorax	Drosophila	68.89 %	No
Histone RNA hairpin-binding protein	drosophila	50.00 %	No
Resistance to inhibitors of cholinesterase protein 3	Nematode	100.00 %	No
Seven B Two (Mammalian 7BT prohormone convertase chaperone) homolog	Nematode	92.42 %	Yes
MUTator	Nematode	62.00 %	No
Dehydrin ERD14	Arabidopsis	100.00 %	No
Dehydrin COR47	Arabidopsis	100.00 %	No
Calvin cycle protein CP12-2, chloroplastic	Arabidopsis	100.00 %	No
Dehydrin ERD10	Arabidopsis	100.00 %	No
Dehydrin Xero 2	Arabidopsis	100.00 %	No
Dehydrin Rab18	Arabidopsis	100.00 %	No
RPM1-interacting protein 4	Arabidopsis	100.00 %	No
Late embryogenesis abundant protein 18	Arabidopsis	100.00 %	No
Late embryogenesis abundant protein 7	Arabidopsis	100.00 %	No
Dehydrin HIRD11	Arabidopsis	100.00 %	No

Late embryogenesis abundant protein 46	Arabidopsis	100.00 %	No
Protein COLD-REGULATED 15A, chloroplastic	Arabidopsis	71.22 %	No
Protein COLD-REGULATED 15B, chloroplastic	Arabidopsis	70.21 %	No
Translocase of chloroplast 159, chloroplastic	Arabidopsis	55.82 %	No
Dehydration-responsive element-binding protein 2A	Arabidopsis	50.15 %	No

* Intrinsically disordered proteins and their disordered contents were retrieved from the DisProt database [2]. Annotations for membrane protein and signal peptide were retrieved from the UniProt database.

Supplementary Table 4. Intrinsically disordered membrane proteins

Protein *	Organism	Disordered content	Ratio of cytoplasmic disorder#	Ratio of non-cytoplamsic disorder§	Length of cytoplamsic portion	Length of non-cytoplasmic portion
T-cell surface glycoprotein CD3 zeta chain	Human	83.54%	100.00%	100.00%	113	9
Prolactin receptor	Human	58.36%	93.41%	0.00%	364	210
CD166 antigen	Human	57.98%	100.00%	62.67%	34	450
Growth hormone receptor	Human	55.02%	94.86%	0.00%	350	246
Isoform A of Reticulon-4	Human	91.36%	99.34%	42.55%	1056	94
Reticulon-4	Human	88.00%	99.34%	0.00%	1056	94
Emerin	Human	73.62%	84.23%	0.00%	222	11
Linker for activation of T-cells family member 1	Human	78.63%	87.66%	0.00%	235	4
Isoform B of Reticulon-4	Human	62.47%	99.15%	0.00%	235	94
Vesicle-associated membrane protein-associated protein B/C	Human	51.44%	56.11%	0.00%	221	0
Potassium voltage-gated channel subfamily E member 1	Human	50.39%	68.25%	46.51%	63	43
Early activation antigen CD69	Human	42.21%	100.00%	16.67%	40	138
Bcl-2-like protein 1	Human	39.48%	44.02%	0.00%	209	7
Integrin beta-6	Human	37.69%	100.00%	31.69%	58	688
T-cell surface glycoprotein CD3 epsilon	Human	31.88%	100.00%	0.00%	55	104

chain						
Stannin	Human	29.55%	45.61%	0.00%	57	10
Adenosine receptor A2a	Human	29.13%	30.93%	0.00%	388	56
B-cell antigen receptor complex-associated protein alpha chain	Human	26.99%	100.00%	0.00%	61	111
T-cell surface glycoprotein CD3 delta chain	Human	26.32%	100.00%	0.00%	45	84
Desmoglein-1	Human	26.31%	57.50%	0.00%	480	525
Isoform 10 of Pro-neuregulin-1, membrane-bound isoform	Human	25.34%	0.00%	25.11%	375	223
High affinity immunoglobulin epsilon receptor subunit gamma	Human	24.42%	38.10%	0.00%	42	5
Ephrin-B2	Human	24.32%	97.59%	0.00%	83	202
Iodotyrosine deiodinase 1	Human	24.22%	0.00%	36.65%	54	191
T-cell surface glycoprotein CD3 gamma chain	Human	24.18%	97.78%	0.00%	45	94
Vesicle-associated membrane protein 2	Human	24.14%	30.11%	0.00%	93	2
B-cell antigen receptor complex-associated protein beta chain	Human	21.40%	100.00%	0.00%	49	131
Receptor tyrosine-protein kinase erbB-2	Human	21.35%	46.21%	0.00%	580	630
Solute carrier family 22 member 17	Human	19.52%	0.00%	54.40%	148	182
Cytochrome b reductase 1	Human	19.58%	52.34%	0.00%	107	53
Delta-like protein 4	Human	19.42%	98.52%	0.00%	135	503

Transient receptor potential cation channel subfamily V member 5	Human	19.07%	27.10%	0.00%	513	86
Antigen peptide transporter 2	Human	18.80%	9.01%	42.19%	433	64
Protein phosphatase 1 regulatory subunit 15A	Human	17.66%	18.14%	0.00%	656	0
Sodium/hydrogen exchanger 1	Human	16.69%	34.26%	0.00%	397	151
Isoform C of Reticulon-4	Human	16.58%	3.15%	0.00%	1046	94
Mitochondrial fission 1 protein	Human	16.45%	1.64%	22.22%	122	9
Proheparin-binding EGF-like growth factor	Human	16.35%	0.00%	24.11%	24	141
Syndecan-4	Human	14.14%	100.00%	0.00%	28	127
Neuroligin-3	Human	13.92%	100.00%	0.00%	118	672
Antigen peptide transporter 1	Human	13.86%	5.89%	24.29%	526	70
T-cell surface glycoprotein CD4	Human	13.32%	100.00%	0.00%	40	371
Cystic fibrosis transmembrane conductance regulator	Human	12.50%	16.49%	0.00%	1122	105
Calcium-binding mitochondrial carrier protein SCaMC-1	Human	10.90%	0.00%	20.39%	112	255
Potassium/sodium hyperpolarization-activated cyclic nucleotide-gated channel 1	Human	10.45%	13.78%	0.00%	675	64
Protein jagged-1	Human	10.26%	100.00%	0.00%	125	1034
Kit ligand	Human	9.89%	0.00%	14.36%	36	188

Apoptosis regulator Bcl-2	Human	9.62%	10.90%	0.00%	211	6
Stimulator of interferon genes protein	Human	9.50%	12.68%	0.00%	284	20
Transient receptor potential cation channel subfamily V member 6	Human	9.28%	12.91%	0.00%	550	57
Neural cell adhesion molecule L1	Human	9.07%	100.00%	0.00%	114	1101
Bone morphogenetic protein receptor type-1A	Human	9.02%	0.00%	37.21%	356	129
Adenosine receptor A1	Human	7.98%	25.74%	0.00%	101	59
C-X-C chemokine receptor type 4	Human	7.39%	0.00%	28.57%	113	91
Disintegrin and metalloproteinase domain-containing protein 10	Human	6.95%	100.00%	0.00%	52	653
Voltage-dependent L-type calcium channel subunit alpha-1S	Human	6.78%	13.12%	0.00%	968	469
T cell receptor gamma constant 1	Human	6.36%	0.00%	7.97%	12	138
Serine protease HTRA2, mitochondrial	Human	6.11%	8.41%	0.00%	333	104
Low-density lipoprotein receptor	Human	6.16%	0.00%	6.91%	49	767
Integrin beta-3	Human	6.09%	100.00%	0.00%	47	692
Band 3 anion transport protein	Human	5.93%	9.85%	0.00%	548	86
Integrin beta-1	Human	5.89%	100.00%	0.00%	47	708
Amyloid-beta precursor protein	Human	5.19%	0.00%	4.39%	48	684
Neurogenic locus notch homolog protein 1	Human	5.13%	16.40%	0.00%	799	1717
Integrin alpha-IIb	Human	4.91%	100.00%	0.52%	20	962

Angiotensin-converting enzyme 2	Human	4.60%	84.09%	0.00%	44	723
Integrin beta-2	Human	4.55%	76.09%	0.00%	46	678
Immunoglobulin alpha Fc receptor	Human	4.53%	0.00%	5.34%	41	206
Histo-blood group ABO system transferase	Human	4.52%	0.00%	5.32%	32	301
TGF-beta receptor type-2	Human	3.70%	0.00%	14.58%	380	144
Autophagy-related protein 9A	Human	3.58%	5.42%	0.00%	554	157
ATP-binding cassette sub-family C member 8	Human	3.54%	5.25%	0.00%	1067	153
Copper-transporting ATPase 1	Human	3.47%	4.57%	0.00%	1139	99
Vascular endothelial growth factor receptor 3	Human	2.71%	0.00%	4.93%	567	751
MHC class I polypeptide-related sequence A	Human	2.61%	0.00%	3.52%	55	284
Mast/stem cell growth factor receptor Kit	Human	2.25%	4.64%	0.00%	431	499
Dystroglycan	Human	1.90%	0.00%	0.00%	120	749
Potassium voltage-gated channel subfamily KQT member 1	Human	1.63%	2.24%	0.00%	490	58
Sortilin	Human	1.44%	0.00%	1.77%	53	678
Mucin-1	Human	1.20%	20.27%	0.00%	74	1135
Epidermal growth factor receptor	Human	1.07%	2.40%	0.00%	542	621
CMRF35-like molecule 1	Mouse	59.05%	94.31%	35.63%	123	174
Vesicle-associated membrane protein 4	Mouse	50.35%	60.17%	0.00%	118	2

Gap junction alpha-4 protein	Mouse	30.33%	59.06%	0.00%	171	73
Gap junction alpha-5 protein	Mouse	29.33%	48.17%	0.00%	218	56
Gap junction gamma-1 protein	Mouse	26.01%	41.20%	0.00%	250	60
Dehydrodolichyl diphosphate synthase complex subunit Nus1	Mouse	24.92%	91.43%	0.00%	70	171
Mu-type opioid receptor	Mouse	22.36%	20.99%	47.22%	181	108
Isoform L of Receptor-type tyrosine-protein phosphatase delta	Mouse	17.99%	0.00%	21.67%	625	1246
Cadherin-1	Mouse	16.86%	98.68%	0.00%	151	686
Plexin-A4	Mouse	16.38%	0.00%	25.56%	636	1213
Glutamate receptor ionotropic, NMDA 2B	Mouse	15.11%	32.99%	0.00%	679	702
Dystroglycan	Mouse	10.86%	0.00%	13.40%	121	724
Exostosin-like 2	Mouse	3.33%	0.00%	3.82%	21	288
Zona pellucida sperm-binding protein 2	Mouse	3.09%	0.00%	3.39%	10	649
Transforming growth factor beta receptor type 3	Mouse	1.76%	0.00%	1.97%	42	763
Neuroligin-2	Mouse	1.67%	0.00%	2.11%	137	664
Vesicle-associated membrane protein 2	Rat	75.86%	93.55%	0.00%	93	2
Vesicle transport through interaction with t-SNAREs homolog 1A	Rat	34.82%	39.20%	0.00%	199	4
Gap junction alpha-1 protein	Rat	30.10%	49.15%	0.00%	234	63
Syntaxin-12	Rat	25.18%	27.71%	0.00%	249	3

Gap junction beta-1 protein	Rat	23.67%	53.17%	0.00%	126	68
Bcl-2-like protein 1	Rat	21.46%	23.92%	0.00%	209	7
Reticulon-4	Rat	15.65%	17.72%	0.00%	1027	94
Small conductance calcium-activated potassium channel protein 2	Rat	13.97%	34.76%	0.00%	233	147
Prestin	Rat	9.81%	19.31%	0.00%	378	114
Gamma-aminobutyric acid type B receptor subunit 1	Rat	8.27%	0.00%	12.35%	164	664
Syntaxin-1A	Rat	7.64%	8.30%	0.00%	265	0
Protein ERGIC-53	Rat	3.68%	0.00%	4.19%	12	454
Amyloid-beta A4 protein	Rat	2.08%	0.00%	2.34%	48	684
Synaptobrevin homolog 1	Yeast	79.49%	98.94%	0.00%	94	6
Synaptobrevin homolog 2	Yeast	79.13%	97.85%	0.00%	93	3
Dolichyl-diphosphooligosaccharide--protein glycosyltransferase subunit 3	Yeast	55.14%	46.15%	73.39%	26	218
Protein OPY2	Yeast	38.89%	0.00%	56.45%	91	248
Protein SSO1	Yeast	25.52%	27.92%	0.00%	265	3
Protein SSO2	Yeast	25.08%	27.51%	0.00%	269	4
Dolichyl-diphosphooligosaccharide--protein glycosyltransferase subunit STT3	Yeast	7.38%	3.92%	7.20%	51	403
Inner nuclear membrane protein HEH2	Yeast	6.33%	13.29%	0.00%	316	326
Cytochrome b-c1 complex subunit Rieske,	Yeast	5.58%	0.00%	8.89%	50	135

mitochondrial						
GDP-Man:Man(3)GlcNAc(2)-PP-Dol alpha-1,2-mannosyltransferase	Yeast	2.55%	0.00%	2.76%	20	507
Potassium voltage-gated channel protein Shaker	drosophila	21.83%	34.05%	0.00%	420	86
Gliotactin	drosophila	21.65%	100.00%	0.00%	207	695
DE-cadherin	drosophila	10.48%	100.00%	0.00%	158	1067
Protein commissureless 1	drosophila	2.97%	5.16%	0.00%	213	136
Resistance to inhibitors of cholinesterase protein 3	nematode	100.00%	100.00%	100.00%	64	272
Cyclic nucleotide-gated cation channel	nematode	29.88%	59.19%	0.00%	370	237
Translocase of chloroplast 159, chloroplastic	Arabidopsis	55.82%	54.71%	100.00%	1466	20
Translocase of chloroplast 132, chloroplastic	Arabidopsis	37.73%	38.53%	0.00%	1181	7
Inner membrane protein ALBINO3, chloroplastic	Arabidopsis	26.84%	61.08%	0.00%	203	98
Protein phosphatase 2C 70	Arabidopsis	2.58%	2.71%	0.00%	553	7

* Intrinsically disordered membrane proteins and their disordered contents were retrieved from the DisProt database [2].

# Ratio of cytoplasmic disorder was calculated by (length of disordered sequence within cytoplasmic portion)/(length of cytoplasmic portion).

ξ Ratio of non-cytoplasmic disorder was calculated by (length of disordered sequence within extracellular or luminal portion)/(length of extracellular or luminal portion).

Supplementary Figure 1

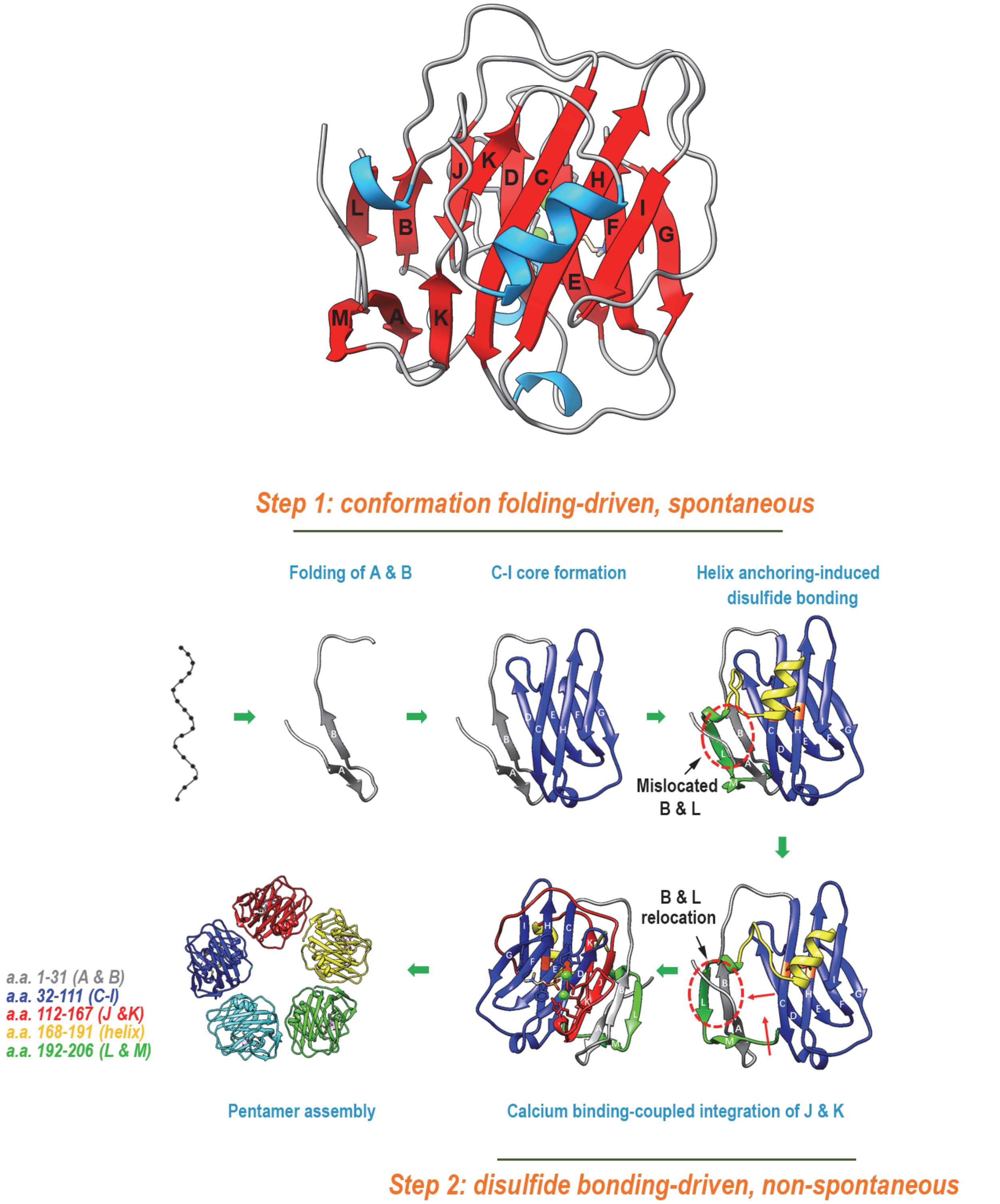

Supplementary Figure 1. The model for native structure formation of CRP. Upper panel: subunit structure of human CRP (PDB 1B09) [3] with all β strands labeled. Lower panel: the folding and assembly of CRP subunits [1]. Step 1 of subunit folding is a spontaneous process: strands A & B folds first, leading to the formation of C-I core; independently folded 168-176 helix then anchors to C-I core covering Cys36 and Cys97, eventually resulting in their bonding. At this step, strands B & L are

mislocated to the edge of C-I core. Step 2 of subunit folding is a non-spontaneous process requiring the assistance of cellular factors. In this process, disulfide bonding evokes conformation changes of C-I core, leading to relocation of strands B & L for integration of strands J & K coordinated by calcium binding. Finally, folded subunits are assembled into the pentamer.

Supplementary Figure 2

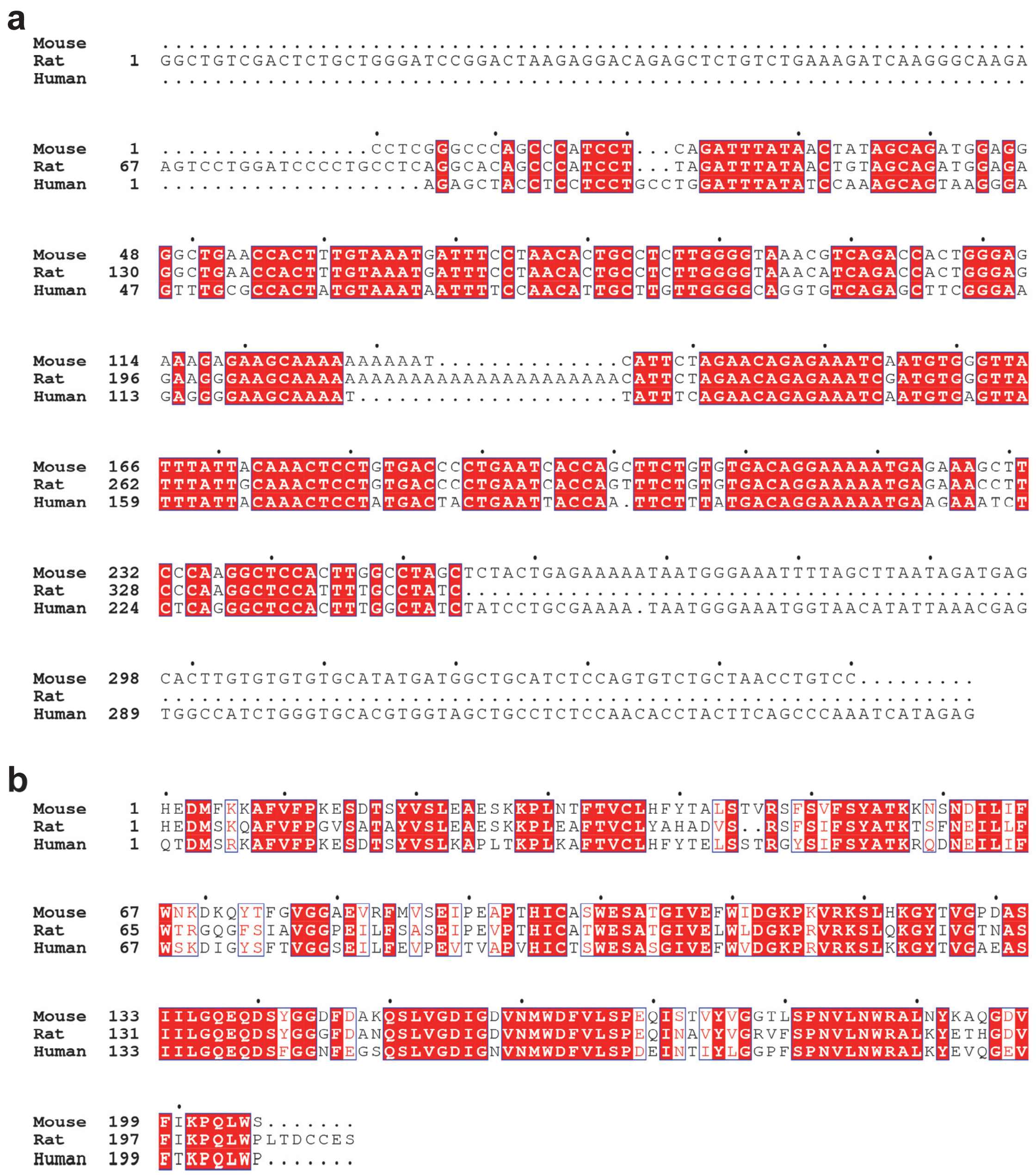

Supplementary Figure 2. Alignment of CRP sequences. Sequence alignment of (a) promoters (0~-350 bp) and (b) amino acids of mouse, rat, and human CRP. Sequences were aligned with ClustalW, and rendered using ESPript [4].

Supplementary References:

1. Lv, J.M. et al. Conformational folding and disulfide bonding drive distinct stages of protein structure formation. *Sci Rep* **8**, 1494 (2018).
2. Hatos, A. et al. DisProt: intrinsic protein disorder annotation in 2020. *Nucleic Acids Res* **48**, D269-D276 (2020).
3. Thompson, D., Pepys, M.B. & Wood, S.P. The physiological structure of human C-reactive protein and its complex with phosphocholine. *Structure* **7**, 169-77 (1999).
4. Robert, X. & Gouet, P. Deciphering key features in protein structures with the new ENDscript server. *Nucleic Acids Res* **42**, W320-4 (2014).
