# Supplementary material for: Secretory quality control constrains functional selection-associated protein structure innovation
Source: Commun Biol. 2022 Mar 25;5:268. doi: 10.1038/s42003-022-03220-3 (PMC8956723; doi:10.1038/s42003-022-03220-3)
Supplement: Supplementary file 8 — Reporting Summary [file 42003_2022_3220_MOESM8_ESM.pdf]

nature portfolio

Corresponding author(s): Yi WU

Last updated by author(s): Feb 16, 2022

Reporting Summary

Nature Portfolio wishes to improve the reproducibility of the work that we publish. This form provides structure for consistency and transparency in reporting. For further information on Nature Portfolio policies, see our Editorial Policies and the Editorial Policy Checklist.

Statistics

For all statistical analyses, confirm that the following items are present in the figure legend, table legend, main text, or Methods section.

n/a	Confirmed	
☐	☒	The exact sample size (n) for each experimental group/condition, given as a discrete number and unit of measurement
☐	☒	A statement on whether measurements were taken from distinct samples or whether the same sample was measured repeatedly
☐	☒	The statistical test(s) used AND whether they are one- or two-sided<br>*Only common tests should be described solely by name; describe more complex techniques in the Methods section.*
☒	☐	A description of all covariates tested
☒	☐	A description of any assumptions or corrections, such as tests of normality and adjustment for multiple comparisons
☒	☐	A full description of the statistical parameters including central tendency (e.g. means) or other basic estimates (e.g. regression coefficient) AND variation (e.g. standard deviation) or associated estimates of uncertainty (e.g. confidence intervals)
☒	☐	For null hypothesis testing, the test statistic (e.g. F, t, r) with confidence intervals, effect sizes, degrees of freedom and P value noted<br>*Give P values as exact values whenever suitable.*
☒	☐	For Bayesian analysis, information on the choice of priors and Markov chain Monte Carlo settings
☒	☐	For hierarchical and complex designs, identification of the appropriate level for tests and full reporting of outcomes
☒	☐	Estimates of effect sizes (e.g. Cohen's d, Pearson's r), indicating how they were calculated

Our web collection on statistics for biologists contains articles on many of the points above.

Software and code

Policy information about availability of computer code

Data collection	N/A
Data analysis	OriginPro 2018C SR1 b9.5.1.195 was used to analyze data.

For manuscripts utilizing custom algorithms or software that are central to the research but not yet described in published literature, software must be made available to editors and reviewers. We strongly encourage code deposition in a community repository (e.g. GitHub). See the Nature Portfolio guidelines for submitting code & software for further information.

Data

Policy information about availability of data

All manuscripts must include a data availability statement. This statement should provide the following information, where applicable:

- Accession codes, unique identifiers, or web links for publicly available datasets
- A description of any restrictions on data availability
- For clinical datasets or third party data, please ensure that the statement adheres to our policy

The crystal structure of human CRP analyzed during the current study are available in the RCSB PDB database (1B09; www.rcsb.org). The sequences of CRP analyzed during the current study were obtained from the UniProt database (www.uniprot.org). The information of intrinsically disordered proteins was retrieved from the DisProt database (www.disprot.org). Single particle datasets that support the findings of this study have been deposited in EMPIAR with accession codes EMPIAR-10960 (mouse CRP), EMPIAR-10959 (rat CRP), and EMPIAR-10958 (human CRP). The authors declare that all other data supporting the findings of this study are available within the article and its supplementary information files.

Field-specific reporting

Please select the one below that is the best fit for your research. If you are not sure, read the appropriate sections before making your selection.

☒ Life sciences ☐ Behavioural & social sciences ☐ Ecological, evolutionary & environmental sciences

For a reference copy of the document with all sections, see nature.com/documents/nr-reporting-summary-flat.pdf

Life sciences study design

All studies must disclose on these points even when the disclosure is negative.

Sample size	Sample-size was determined empirically.
Data exclusions	No data were excluded.
Replication	Reproducibility of the experimental findings were verified by at least 3 independent experiments with 2-3 technical replicates or at least 5 animals.
Randomization	Randomly numbered animals were allocated into control and treatment groups according to numeric parity.
Blinding	N/A

Reporting for specific materials, systems and methods

We require information from authors about some types of materials, experimental systems and methods used in many studies. Here, indicate whether each material, system or method listed is relevant to your study. If you are not sure if a list item applies to your research, read the appropriate section before selecting a response.

Materials & experimental systems

n/a	Involved in the study	
☐	☒	Antibodies
☐	☒	Eukaryotic cell lines
☒	☐	Palaeontology and archaeology
☐	☒	Animals and other organisms
☒	☐	Human research participants
☒	☐	Clinical data
☒	☐	Dual use research of concern

Methods

n/a	Involved in the study	
☒	☐	ChIP-seq
☒	☐	Flow cytometry
☒	☐	MRI-based neuroimaging

Antibodies

Antibodies used

Primary antibodies: HRP-labeled anti-His antibody (Proteintech, Rosemont, IL; catalog number: 66005-I-Ig; lot number: 10004365)
Antibody dilution ratio: 1: 10000 for ELISA

Primary antibody: Anti-strep mAb (Sangon Biotech, Shanghai, China; catalog number: D195307)
Antibody dilution ratio: 1:30000 for WB and 1:3000 for ELISA

Primary antibody: 1D6 mAb
Antibody dilution ratio: 1: 200 for ELISA

Primary antibody: 3H12 mAb
Antibody dilution ratio: 1: 1000 for WB and 1:200 for ELISA

Primary antibody: Sheep-anti-human CRP polyclonal antibody (BindingSite; catalog number: DX044; lot number: 445029-1)
Antibody dilution ratio: 5 μg/ml for ELISA

Secondary antibody: HRP-labeled Goat Anti-Mouse IgG (H + L) (Jackson ImmunoResearch, West Grove, PA; Catalog number: 115-035-003; Lot number: 125229);
Antibody dilution ratio: 1: 40000 for WB and 1:4000 for ELISA

Validation

Commercial antibodies were validated by the manufacturers. Custom antibodies were validated by Dr. Potempa and our previous studies.

Eukaryotic cell lines

Policy information about cell lines

Cell line source(s)	COS-7 cells were obtained from cell bank of Chinese Academy of Sciences (Shanghai, China).
Authentication	COS-7 cells were authenticated by the supplier.
Mycoplasma contamination	This cell line was tested negative for mycoplasma contamination
Commonly misidentified lines (See ICLAC register)	N/A

Animals and other organisms

Policy information about studies involving animals; ARRIVE guidelines recommended for reporting animal research

Laboratory animals	Male and female C57BL/6 mice of 8~9 weeks; Male SD rats of 8~9 weeks.
Wild animals	N/A
Field-collected samples	N/A
Ethics oversight	All experiments were conducted according to the protocols approved by the Ethics Committee of Animal Experiments of Xi'an Jiaotong University.

Note that full information on the approval of the study protocol must also be provided in the manuscript.
